# Remarks upon the Subject of Infanticide

**Published:** 1832-11

**Authors:** Alfred S. Taylor

**Affiliations:** Lecturer on Medical Jurisprudence, &c. in Guy's Hospital.


THE LONDON

### Medical and Physical Journal.

405, VOL. LXVIII.] NOVEMBER 1832. [77, *New Series*.

"For many fortunate discoveries in medicine, and for the detection of numerous errors, the world is indebted to the rapid circulation of Monthly Journals; and there never existed any work to which the Faculty in Europe and America, were under deeper obligations than to the 'Medical and Physical Journal of London,' now forming a long but an invaluable series."—RUSH.

### ORIGINAL PAPERS.

### INFANTICIDE.

*Remarks upon the Subject of Infanticide.* By ALFRED S. TAYLOR, Esq., Lecturer on Medical Jurisprudence, &c. in Guy's Hospital.

THE frequency with which cases of infanticide come before our courts of justice must have been remarked by every member of the profession who has paid the least attention to the progress of the science of medical jurisprudence; but, in spite of the stimulus to inquiry which this condition of things may be supposed to have brought about, it is questionable whether the medical evidence ordinarily elicited on these occasions has sustained any material improvement since the time of the celebrated Dr. HUNTER. It is well known that this physician, by the publication of a small tract, "on the Uncertainty of the Signs of Murder in Bastard Children," succeeded in overthrowing almost entirely the established opinions of the profession regarding the proofs or tests employed upon these occasions. The influence which his name and authority have had in giving rise to this result has not been confined to the medical profession only: judges and juries have been carried away by the same prejudice; convictions have been recently as rare as they formerly were frequent; and he who now peruses the reports of trials for this detestable crime is almost inevitably led to the conclusion that no evidence can be offered sufficient to establish the fact of its commission; or, if offered, that it will not be received by those who administer the law. In the mean time the sanguinary statute which prevailed in this country at the period at which Dr. Hunter wrote has been repealed,* and

* Stat. 21 Jac. I. c. 27. By this statute it was enacted, that if any woman, concealing the birth of her bastard child, was suspected of having murdered it, she was to suffer death, as in other cases of murder, unless she could prove by one witness that it was born dead!

the crime of infanticide is now required to be proved by the same rules of evidence as those which are received with regard to murder.

Those who have perused the tract of Dr. Hunter just now alluded to, cannot fail to have perceived that the spirit by which the author was actuated was rather that of humanity than the rigid investigation of truth. In every page do we meet with passages which appeal to our feelings, rather than to our judgment; in every sentiment do we meet with some reflection pointing out the admitted defects of the tests and proofs commonly resorted to, rather than those instances in which they may be safely relied on. If his principle had been that of doing away with the utility or value of all medical evidence in such cases, he could not have pursued a course better adapted for the accomplishment of this object. He has thrown out general objections to the employment of the hydrostatic test, many of which are more imaginary than real; while others, which have the appearance of being founded in truth, are unsupported by legitimate experimental researches. Notwithstanding, however, the superficial manner in which these objections are framed, they have sufficed to discourage all further investigations on the subject in the greater number of the profession, and to create in our courts of law that species of scepticism, with regard to this branch of medical evidence, which strips it of all claim to admission. I have heard it remarked by experienced barristers, that there is nothing more easy than to set aside the evidence of the medical witness in cases of infanticide, where that evidence bears upon the proofs required to support the vitality of the child; and the following case, which is of recent occurrence, may perhaps be adduced in support of this statement.

On the last northern circuit, a female was tried before Mr. Justice Parke, on the charge of child-murder.

The prisoner, it appeared, left her home on the 7th of February last, to go to the house of a relative in a neighbouring county. She was far advanced in pregnancy, but did not appear unwell, although she had sent for medicine. About a week after her arrival, she left the house, and remained absent for some time, as it was supposed, walking. She returned home that evening, and appeared to be much indisposed; but no particular attention was paid to this circumstance. About five days after this, a labouring man, who was employed at an adjoining farm, while passing along the bank of a river that runs near the house in which the prisoner was staying, discovered something floating in the water, which, upon being drawn out, proved to be the body of a full-grown male child. The body was subsequently examined by a surgeon, who, at the coroner's inquest, deposed that there were certain appearances about it which led him to conclude that the child had been born alive. The prisoner was accordingly arrested, and committed on the charge of child-murder.

On the trial, the general evidence, as described, having been gone through, and it having been stated, in addition, by one of the witnesses, that the prisoner had confessed she had been delivered of a child, but that this child was dead at the birth, the opinion of the medical witness as to the fact of the child having been born alive was called for. He declared that the appearances of the thoracic viscera favored the belief in the affirmative; but he at the same time admitted, in cross-examination, that the child might not have been born with life; also, that a child might breathe, and the blood might circulate, yet still it might be impossible to preserve its existence. Upon this, the jury acquitted the prisoner of the charge of murder, and found her guilty of the concealment of birth.

This is not a solitary instance. It may have been noticed that, on the circuits, for several years past, the charge of infanticide has repeatedly fallen to the ground, because the medical practitioner was unable to throw any light upon that question which is always raised by the court, as to the vitality of the child. Now it is not my wish to be identified with those who are constantly crying out for the infliction of capital punishment, or who would desire to see a woman condemned upon unsatisfactory evidence; but let it be remembered that it is not humanity, but an injury to the community, to enact laws, and then allow them to be violated by neglecting to adopt the most efficient means required for their enforcement. Our code is sufficiently Draconic, and, it is true, if convictions were more frequent in cases of infanticide, more victims would be brought to the scaffold: but this is a question for the legislator, and not for the physician, to determine. If, by experience, it be found that capital punishment is insufficient to check the progress of the crime, let the law be altered; but let it always be rigidly enforced. As it now stands, it may probably appear too severe in the eyes of jurymen; and hence they are ready to cling to any deficiency in the medical evidence, in order to acquit the prisoner, or at least to save her from the gallows. If it were otherwise, it would be impossible to explain why, out of twelve such charges, more than two thirds should end in acquittal, where nothing seems to be wanting in the chain of evidence but the proof of the vitality of the child.

It has unfortunately resulted from the tract of Dr. Hunter, that many have been deterred from prosecuting inquiries on this interesting subject, by the bold assertions which he has made regarding the inefficiency of the *hydrostatic test.* Many, who would have been otherwise disposed to examine fairly the objections, have been seduced by the name of the author, have taken his word for their validity, and have been disposed to throw ridicule upon those who should pretend to mistrust his statements. There is no doubt that the "test," as it has been called, is in some degree liable to lead to erroneous conclusions, where sufficient care is not taken to attend to all the particular conditions required for its employment; but, because it *may* lead, in the hands of the inexperienced, to erroneous conclusions, it is not, therefore, to be utterly discarded from practice, since, by the adoption of such a principle, we should be guilty of the greatest inconsistency. With regard to the tests employed for the detection of arsenic, or other mineral poisons, in mixtures of animal and vegetable substances, there is, perhaps, no individual in the profession who is not aware of the numerous fallacies which may arise and defeat the investigation; fallacies so striking that, even in simple solutions, the results require to be corroborated by other experiments of a negative character. Yet, with the knowledge of this fact, scarcely any attempts have been made to shew that the objections urged to the employment of the hydrostatic test, in cases of infanticide, might be set aside by the adoption of counter-experiments; while those few who have made the attempts have been ridiculed, as undertaking a vain and fruitless labour. It has not yet been shewn that the pulmonary test is more likely to lead to mischievous inferences in questions of infanticide, than is the injudicious employment of the tests for arsenic in questions of suspected poisoning by that substance. Errors in either case are equally to be dreaded; but where the investigation is undertaken by, or entrusted to, one but little prepared for the task, it is surely neither just nor reasonable to throw all the blame upon the means employed, and not upon the individual who employs them.

Sufficient has been said to shew the influence of Dr. Hunter's name over our own profession: if any further evidence of the fact were demanded, it might readily be obtained by referring to the reports of trials that have taken place since the period at which he wrote. It is now, however, my object to shew that this influence has extended itself even over our highest judicial authorities, and that the test has been as loudly denounced from the bench as in the witness box. The following are the remarks made by Lord Lyndhurst on the case of Maria Warrington, indicted for child-murder at the Nottinghamshire lent assizes, in 1831.

After adverting to the declarations made by the prisoner, his lordship, in his charge to the grand jury, called their attention to the second point of the case, namely, the fact of the child having been born alive. "We are much indebted, said the learned judge, to the exertions of the justly-celebrated Dr. William Hunter, for leading us out of the great error into which nearly all the medical faculty had fallen upon this subject. The prevailing opinion was that, if the lungs would float in water, it was to be considered conclusive that the child was born alive; but Dr. Hunter's tract has proved the fallacy of such a position, and courts and juries are not justified in finding verdicts upon such evidence alone. In this case, the evidence which will be placed before you will be entirely of that nature; and I think *you will not be safe in coming to a conclusion unfavorable to the prisoner from such evidence*."*

The woman was acquitted of the capital charge, but found guilty of concealment!

By this it will be perceived that the opinions of Dr. Hunter, given fifty years ago, at a time when medical jurisprudence was unknown in this country, are held up as the standard according to which the verdict of the jury is to be framed. This is but too frequently the case on trials for this offence; if it proceed not directly from the bench, it is not forgotten by the counsel for the prisoner; and it must be confessed that the cross-examination of the medical witness but too frequently warrants these sentiments of scepticism and distrust. Since it is allowed by all, that if any proofs of vitality can be brought forward on these occasions, they must be found principally, if not solely, in the organs of circulation and respiration, it becomes a question of importance to determine how far the objections raised by Dr. Hunter are entitled to credit. As long as it is required by law, or by the opinions of our great legal authorities, that the child must be proved *to have breathed after birth*, preparatory to the charge of murder being established, it is obvious that, if we abandon the proofs derived from the state of these organs, because they do not *always* assist us, we are virtually resigning that which can alone lead to the conviction of an offender;

* Medical Gazette, vol. viii. p. 156.

we are declaring that medical science can afford no solution of the question at issue; and hence we bring about the acquittal of the guilty, as well as the innocent. Unless, therefore, a newly-born child has been *seen* to breathe, there can be but little hope of substantiating a charge of murder, however atrocious may be the circumstances of the case, or however strong the general evidence against the prisoner.

It is assumed by those who contend for the employment of the *docimasia pulmonaris,* or hydrostatic test, that where the lungs of the infant have been distended by the act of respiration, they become buoyant, and float when placed in water: where, however, the act of respiration has not been *perfectly* accomplished, this result is not to be expected; the lungs are then of equal density with the liver, and sink to the bottom of the vessel when immersed in water. These, then, are two general propositions, upon which the whole evidence in favor of the pulmonary test is based, and by which it must finally stand or fall. Now, it is not maintained by medical jurists, that the lungs of the fœtus may not float or sink from the operation of other causes ; and yet the whole of the objections of Dr. Hunter and others are grounded upon this imaginary difficulty.* If any individual were to stand forward and say that the silver *test* should not be employed in the examination of suspected solutions of arsenic, because a yellow precipitate may be obtained where it is added to certain other substances not of a deleterious nature; or that the copper and sulphuretted hydrogen *tests* should, under like circumstances, be abandoned, they would not be the less employed or relied on by the careful experimentalist, since he is prepared to meet, and generally able to guard against, those fallacies which to the inexperienced might seem insurmountable. I do not wish to be understood as saying, that the docimasia pulmonaris, in regard to cases of infanticide, will afford, in every case, such certain and satisfactory results as will the above-named tests for arsenic in suspected solutions of that poison; but it should not be forgotten, that it is only recently that these have been brought to their present degree of perfection; and that it is but a few years since the copper and silver tests were discarded as useless in examining solutions of arsenic. Even now they will not serve us in every case; and it not unfrequently happens that the fact of poisoning is most clearly proved by the

* There are but very few writers on medical jurisprudence in the present day, who have experimented upon this subject, who do not admit that this test may be of the most important service in investigations relative to infanticide, where those precautions are observed which will be pointed out in the sequel.

general evidence, as well as by the symptoms and morbid appearances, when none of our tests will afford us the least evidence of poison in the fluids taken from the viscera. On the other hand, a poison may be found in the fluids of the alimentary canal, and be proved to be such by the application of our tests; but, although this affords presumptive, it does not yield *positive* evidence that the individual has died from that poison: for, as has happened in more than one instance, it may have been introduced for some diabolical purpose after dissolution. It is here, as in every other subject appertaining to medical jurisprudence, we must proceed with caution in drawing our inferences: we must carefully estimate the fallacies to which all medical evidence is liable, and attribute no more to the means of our investigation than is consistent with sound physiological and pathological reasoning. The corollary to be drawn from these observations is clear. Our tests will fail to discover poison where it has indubitably been the cause of death, they will detect its presence where death may have taken place from other causes: and, in the hands of the inexperienced, they may lead to the most pernicious results. If, therefore, we were inclined to remain content with indicating this "uncertainty" in the "signs" of poisoning, without extending our views to more than one side of the question, as has been done with regard to the subject of infanticide, we should have but little cause to congratulate ourselves upon the important discoveries recently made in the department of toxicology.

It will now be necessary to call the attention of the reader to the mode of investigation pursued by Dr. Hunter. After having alluded to the mistakes that might arise respecting those appearances in the head of the newly-born child which simulate marks of violence, he comes to the material question, viz. "in suspicious cases, how far may we conclude that the child was born alive, *and probably murdered by its mother,* if the lungs swim in water?" This sentence, it will be perceived, contains a remarkable passage, upon which it will be proper to make a remark before proceeding further. The question of murder is here connected, in a most extraordinary manner, with the question relating to the vitality of the child; as if, because the child were proved to have been born alive, it must necessarily have been murdered. This anomaly in reasoning, however, must not be attributed to any error on the part of Dr. Hunter, although he, perhaps, as a physiologist, and as one whose opinions were likely to have a powerful influence on the minds of the profession, ought not to have mixed up this appeal to the feelings in the discussion of a purely physiological question, but to the particular construction of the law (21 Jac. I. c. 27,) which prevailed in his time. By this, contrary to the principles of humanity and justice, the *onus probandi* of the whole case was generally thrown upon the unfortunate female who was the subject of the charge; for, if it were proved that she had concealed the birth of her child, and if she were unable to procure a witness who could swear that the child was born dead, she was held to be guilty of the murder, and punished accordingly.* Where, therefore, it was shewn by the medical evidence that the child had probably lived to respire, it was almost tantamount to the condemnation of the mother; for no account was taken of the possibility of the death of the child having proceeded from accidental causes. So far, then, it will be perceived Dr. Hunter was justified in connecting the question of murder with the question of vitality; although the former ought to have been left to the decision of the court, while the latter should have exclusively occupied the attention of the physician.

However applicable this general proposition may have been fifty years since, it can now no longer be entertained; a point which seems to have been most unaccountably lost sight of by the opponents of the Docimasia pulmonaris. Thus, as our law is at present administered, the fact of the child having lived to respire is proved by one species of evidence; while the question as to whether it have been murdered or not after its birth, requires a totally distinct series of proofs, upon the value of which it is for the jury, and not for the medical jurist, to determine. I must not, however, omit to state that, unless the child can be proved to have lived (which, in the legal sense, signifies to have respired,) after its birth, the question of murder cannot be entered into, and, therefore, the prisoner must be acquitted. In thus rigidly insisting that the act of respiration should be proved to have been performed, prior to a charge of murder being entertained, our legislators seem to have forgotten that a child may be born alive, and not respire, and yet be wilfully destroyed by the advice or collusion of the mother:† or, again, that its life may be taken away

* Another remarkable feature in this statute was, that it limited the commission of the crime to the mothers of *illegitimate* children only, as if it were not equally criminal in a married as in an unmarried female to destroy her offspring. The law, as it at present stands, (9 Geo. IV. c. 31,) makes no distinction of persons; and, therefore, the *legitimacy* of the offspring does not make the offence less atrocious.

† A child may be born enclosed by its membranes, and live for some minutes without respiring, if we are to believe the testimony of Wrisberg; and, again, while it is yet in the passage, and before it can have completed the act of respiration. It may be said that these are imaginary cases; but it is by no means improbable that they are of very frequent occurrence; and the reason why they have never been made a subject of public exposure is very obvious. If, in every charge of infanticide, the absence of any of the ordinary changes produced in the lungs by the respiratory process be considered, in our courts of law, as sufficient to put a stop to all legal proceedings against the prisoner on the capital offence, it is not likely that any such cases can come to light.

"If the lungs float in water, we may, says Dr. Hunter, be assured that they contain air; but we are then to find out if that air be generated by putrefaction." The means to be adopted for determining this important question, that is to say, whether the air be the product of putrefaction or whether it have resulted from the commencement of the process of respiration, are then loosely stated. It is a well-known fact that the lungs, like other soft parts of the body, are liable to decomposition, during which various gases are extricated; and these, by distending the texture of the organs, render them so buoyant that they will readily float if placed on water. But it must be evident that this objection is only of a partial nature; for it cannot hold in a very large proportion of cases, and even where its validity is admitted it will be seen that there are means to be adopted by which the careful experimentalist may guard against any liability to fallacy in the results.

An attempt has been made by some writers on legal medicine to shew that the lungs, of all the softer textures of the body, are less liable to putrefaction than any of the other organs; and hence it has been argued, that the body of a still-born fœtus may be in a partial state of putrefaction, while the lungs will sink in water and indicate that respiration has not taken place. I do not know that any great stress is to be laid upon such statements; for, as far as my own observation extends, the results of experiments upon this subject will vary greatly according to the season of the year at which they may be performed. It is at least certain that the lungs are much less liable to be rendered buoyant by the process of putrefaction, when entire and contained within the cavity of the thorax, than when divided into small pieces and removed it may be born in a state of asphyxia, still capable of resuscitation, provided proper measures be adopted; the neglect of which must surely constitute the offence of infanticide by omission.

from this cavity, to be exposed to the influence of air and water. Even here, however, we occasionally meet with the most conflicting results; but in order more fully to elucidate the question, I will mention one or two experiments.

In the month of February last I divided the lungs of a stillborn fœtus, about forty-eight hours after delivery, into sixteen pieces, and placed them in a vessel of water in a room, the mean temperature of which was about 40°. I allowed them to remain for a period of *fifty days*, at the end of which time, although their texture had become softened by decomposition, they were still lying at the bottom of the vessel. There was not a single divided portion which had acquired sufficient air to render it in the least degree buoyant.

In another experiment, performed in March, I divided the lungs into a still greater number of pieces, and exposed them in a similar manner to a mean temperature of 50° for sixty-eight days, at the end of which time they still covered the bottom of the vessel, having become converted into a thick reddish-coloured semifluid mass.

Having been called upon, during the month of June, to investigate a supposed case of infanticide, and having ascertained that the child had not respired, I took the opportunity of repeating my former experiments upon its lungs. In the first place, I may state that the subject in this case had been dead five or six days, and the body was already in a state of decomposition; but, notwithstanding this circumstance, the lungs were comparatively fresh, presenting that appearance which they usually do in a stillborn fœtus, and they sank in water when immersed entire or divided. Having allowed the divided portions of the organs to remain in the water, I found, at the end of five days, that the greater number had risen to the surface, the temperature during this time varying from 60° to 70°. An experiment, the counterpart of this, which was undertaken by M. Belliard in the year 1826, was attended with different results. This physiologist divided the lungs of four stillborn fœtuses into a number of pieces, and allowed them to remain in water from the 18th June to the 25th August, at the end of which time, although the putrefactive process had proceeded very far, not one of the divided portions had risen to the surface; thus proving that there are some conditions regarding the generation of air in the lungs during putrefaction with which we are unacquainted.*

What conclusions, then, are we justified in drawing from the preceding observations?

* Orfila, Médecine Légale, tom. i.; art. Infanticide.

1st. That the objection raised against the employment of the hydrostatic test, upon the ground of the lungs being rendered buoyant by the process of putrefaction, can only hold with regard to a limited number of cases.*

2d. That the body of a stillborn fœtus may become partially decomposed, while the lungs will resist this process, especially where these organs are retained within their proper cavity.

3d. That the lungs of a stillborn fœtus may be exposed for a considerable time to those causes which favor the putrefactive process; that they may become entirely decomposed; while, notwithstanding all these conditions, they will not acquire sufficient air to render them buoyant.

But we will now presume that the results of all experiments made with the lungs of stillborn infants will be similar to those of the third experiment detailed above; that is to say, that, after the lapse of five or six days' exposure, these organs will become buoyant through putrefaction, and float. We have, then, to inquire whether there be no means by which the distention of texture thus produced by putrefaction is to be distinguished from that natural change in the organs that ensues from the establishment of the process of respiration.

Dr. Hunter says,† "to determine this question we are to examine the other internal parts, to see if they be emphysematous or contain air, and we must examine the appearance of the air-bubbles in the lungs with particular attention. If the air which is in them be that of respiration, the air-bubbles will hardly be visible to the naked eye; but, if the air-bubbles be large, or if they run in lines along the fissures between the component lobuli of the lungs, the air is certainly emphysematous, and not air which has been taken in by breathing."

There can be no doubt of the truth of these observations, but they are given in too concise a form to do away, to any extent, with the prejudice which the previous objection has excited. The diagnosis is but feebly drawn; and, unless we can call to our aid some other means of research, we are not likely to remove the difficulty. In all the cases of doubt which I have met with, the disposition of air in the pulmonary structure has certainly assisted me greatly in coming to

* Out of nine cases of suspected infanticide which have fallen under my own observation, within a very recent period, I have only met with one instance in which the effects of putrefaction threw some degree of obscurity over the results. The particulars of this case will be detailed hereafter.

† "On the Uncertainty of the Signs of Murder in the Case of Bastard Children," p. 29.

a conclusion as to its origin. We ordinarily meet with vesicles of considerable size, where the air is derived from putrefaction; and it is commonly situated superficially, although this is not so necessary a condition as Professor BECK and others have supposed; for the precise seat of the air will depend on the degree of putrefaction, the situation in which the child is found, the season of the year, and other circumstances of the like nature.

One of the most important diagnostic signs recommended for the solution of this question, is that which depends upon the results obtained by a forcible compression of these organs. Thus it is affirmed that, if the air be derived from putrefaction, it may be expelled from the organs without any difficulty, by employing a moderate degree of compression; whereas, on the contrary, where it is derived from respiration, no compression short of the utter disorganization of the lungs will cause the buoyant portions to sink. In a general way, this proposition may be considered as correct: for where putrefaction is but little advanced, it will not be difficult to rupture the superficial vesicles containing the air, and therefore to cause the portions of lung to sink. This I have verified repeatedly by experiment, and there is scarcely a writer on forensic medicine who has not reported similar results from his own observations. But the question now presents itself, can the air generated by putrefaction in the lungs of a stillborn child be at all times, and under all circumstances, forced out by compression? I do not think that question has been as yet satisfactorily answered in the affirmative, although Professor MARC and others have fully admitted it as a most certain discriminating sign.* As far as the experiments which I have made upon the lungs of stillborn fœtuses extend, I have never met with any difficulty in disengaging the air arising from the process of decomposition: but I have very recently met with an instance, in making an inspection of a fœtus, the history of which was unknown, wherein it was impossible to come to any satisfactory decision upon this important point.

The child in question had been taken out of a watercloset, in which, from all appearances, it had remained a considerable time. I judged it to be a seven-months' child. The whole surface of the body was completely discoloured by the putrefactive process; the eyes had disappeared from the orbits: the features were scarcely distinguishable, and the skin had acquired in some measure the toughness and elasticity of leather.

* Dictionnaire des Sciences Médicales; art. Docimasie Pulmonaire.

On opening the abdomen, a large quantity of gas escaped, and the viscera were in such a state of decomposition, that all floated when immersed in water, with the exception of the spleen. On removing the anterior part of the parietes of the thorax, the lungs became immediately pushed forwards by the elastic tension of the gases contained within their structure, to such a degree that they appeared to be three times their ordinary size, and completely concealed the other viscera. The pericardium was distended like a bladder, and the heart itself, when removed and placed in a vessel of water, floated. The foramen ovale was not in the least degree closed; it had precisely the appearance which it has before the establishment of respiration. The canalis arteriosus was pervious, and of equal diameter throughout its whole course. The lungs were highly putrefied; they had a dark colour, and were slightly congested behind; they readily floated when immersed in water, both entire as well as when divided into small pieces. The central portions floated as readily as those taken from the exterior, proving that, whatever were the cause of their distention, it was not of a partial nature, but had operated equally throughout their whole structure. The air was collected in vesicles, the greater number of which were distinctly visible to the naked eye; and this so far warranted the idea that it had resulted from putrefaction. The divided portions were now successively subjected to violent compression; but, notwithstanding the repeated efforts made to extricate the air, we could only cause two of the portions, out of the whole number, to sink. The remaining portions, although considerably reduced in bulk, still retained sufficient of the elastic fluid by which they were distended to yield a distinct feeling of crepitation, and to enable them to remain upon the surface of the water. Whence, then, came this air? Its appearance and diffusion throughout the organs tended to favor the supposition that it had originated in the process of putrefaction: at the same time, the state of the foramen ovale and of the canalis arteriosus did not allow me to infer that the respiratory process had been for any length of time continued, supposing it to have taken place at all. The umbilical cord was too much decomposed to afford any corroborating presumption; and thus, although it was probable that the child had not respired, there was no appearance which could assist us in determining the question with positive certainty. It may be said, that the impossibility of expelling the air which existed in the organs, proves that it was introduced by the respiratory process; yet this, be it remembered, is the *petitio prin-*

*cipii* of the whole investigation. If it could once be fairly shewn, by a series of well-conducted experiments, performed on subjects at various ages, and under every condition which we may suppose to influence the progress of putrefaction, that the view taken by Professor Marc was correct, we might admit his conclusions; but, until this takes place, we must be guided by common observation.

We have now to examine the converse of our proposition; namely, whether air, introduced by the act of respiration, cannot be expelled by compression? Where this process has been fully and perfectly performed, it is not probable that any degree of compression will suffice to deprive any portion of a healthy lung of its air, or cause it to sink. The question, however, may be raised with regard to fœtuses that have not attained to the full period, and have only survived their birth a few hours; in which cases it is observed that the pulmonary organs are sometimes not sufficiently distended to enable them to float on water, although, when divided, some internal portion may be found to be sufficiently buoyant to rise to the surface; more especially, according to the observations of Dr. Hutchinson, in the right lung.* No writer on the subject appears to have directed his attention to this particular point. It has been asserted, generally, that the air cannot be expelled by compression where it is derived from respiration; yet no experiments are brought forward in support of this assertion, with regard to subjects whose respiration may have been but feeble and imperfect. I have not yet been enabled to procure fœtuses fitted for the performance of such experiments; but it does not appear improbable that the small quantity of air may be as readily forced out in these cases as if it were artificially introduced, or a simple result of putrefaction. Should this be subsequently proved, it will be impossible to determine whether such a fœtus, when met with in a state of decomposition, have respired or not; although, where the body is not decomposed, it is clear either that the child must have respired or that the lungs must have been artificially inflated.

We are, then, here met by two difficulties: it is doubtful, first, whether the air generated by putrefaction can in all cases be forced out; and, second, whether the air introduced by respiration may not be in some instances expelled from the lungs. But the knowledge of the conditions under which these difficulties arise divests them of that tendency to cause those errors in our decisions which some have so loudly

* Hutchinson on Infanticide.

maintained. With regard to the first, it can only be met with in subjects in which putrefaction is very far advanced; and, when it does occur, it may be allowed to have its full value in favor of the party accused, for it can never militate against her.* The same may be said of the second; for the only error into which the examiner could fall would be that of pronouncing a child, which had survived its birth but a short time, to have been stillborn; an error which will still be in favor of the party accused, and which, perhaps, in the present state of our knowledge, it is impossible for human ingenuity to avoid.

I trust, then, I have made it sufficiently obvious that the objection on the ground of "putrefaction," when considered in all its bearings, is nugatory; that is to say, with regard to cases in which the considerate witness will think it his duty to give evidence. It is, I repeat, greatly to be regretted that Dr. Hunter should only have enunciated the objection, without pursuing the subject through all its ramifications: there is no doubt that, if he were now living, he would be among the first to regret the influence which his name and authority have had in carrying a prejudice through the minds of lawyers and physicians, against one of the most important sources of evidence in cases of infanticide. The object for which he wrote, namely, the removal of the sanguinary effects of a statute framed in a barbarous age, no longer exists. The law of infanticide and the law of homicide are now one and the same, although the general feeling in favor of his views is yet universal. Let us, however, hope that the spirit for observation which is at present diffusing itself through the minds of all classes will do away with all scepticism and credulity on the subject, and that physiologists and jurists will truly appreciate the valuable application of those signs in cases of infanticide, which it has been hitherto too much the custom to despise and condemn.

(To be continued.) vol. lxix. p. 1.

35, *Great Marlborough street; October* 1832.

* Where the foramen ovale, canalis arteriosus, and umbilical cord, do not afford strong evidences that circulation and respiration have taken place, and where, at the same time, the body is considerably *decomposed*, no prudent medical witness would assert that the mere floating of the lungs, and the impossibility of making them sink by compression, were signs sufficient to determine the question of the vitality of the child, according to the meaning put upon this term by the law.

